# Hyperspectral Technique Combined With Deep Learning Algorithm for Prediction of Phenotyping Traits in Lettuce

**DOI:** 10.3389/fpls.2022.927832

**Published:** 2022-06-30

**Authors:** Shuan Yu, Jiangchuan Fan, Xianju Lu, Weiliang Wen, Song Shao, Xinyu Guo, Chunjiang Zhao

**Affiliations:** ^1^National Engineering Research Center for Agro-Ecological Big Data Analysis and Application, Anhui University, Hefei, China; ^2^Beijing Key Laboratory of Digital Plant, China National Engineering Research Center for Information Technology in Agriculture, Beijing, China

**Keywords:** plant phenotyping, hyperspectral imaging, deep learning, lettuce, SSC, pH

## Abstract

The currently available methods for evaluating most biochemical traits of plant phenotyping are destructive and have extremely low throughput. However, hyperspectral techniques can non-destructively obtain the spectral reflectance characteristics of plants, which can provide abundant biophysical and biochemical information. Therefore, plant spectra combined with machine learning algorithms can be used to predict plant phenotyping traits. However, the raw spectral reflectance characteristics contain noise and redundant information, thus can easily affect the robustness of the models developed *via* multivariate analysis methods. In this study, two end-to-end deep learning models were developed based on 2D convolutional neural networks (2DCNN) and fully connected neural networks (FCNN; Deep2D and DeepFC, respectively) to rapidly and non-destructively predict the phenotyping traits of lettuces from spectral reflectance. Three linear and two nonlinear multivariate analysis methods were used to develop models to weigh the performance of the deep learning models. The models based on multivariate analysis methods require a series of manual feature extractions, such as pretreatment and wavelength selection, while the proposed models can automatically extract the features in relation to phenotyping traits. A visible near-infrared hyperspectral camera was used to image lettuce plants growing in the field, and the spectra extracted from the images were used to train the network. The proposed models achieved good performance with a determination coefficient of prediction ( $R_{p}^{2}$ ) of 0.9030 and 0.8490 using Deep2D for soluble solids content and DeepFC for pH, respectively. The performance of the deep learning models was compared with five multivariate analysis method. The quantitative analysis showed that the deep learning models had higher $R_{p}^{2}$ than all the multivariate analysis methods, indicating better performance. Also, wavelength selection and different pretreatment methods had different effects on different multivariate analysis methods, and the selection of appropriate multivariate analysis methods and pretreatment methods increased more time and computational cost. Unlike multivariate analysis methods, the proposed deep learning models did not require any pretreatment or dimensionality reduction and thus are more suitable for application in high-throughput plant phenotyping platforms. These results indicate that the deep learning models can better predict phenotyping traits of plants using spectral reflectance.

## Introduction

Plant phenotyping is an interdisciplinary field of research that collects and analyses plant phenotyping traits, such as biophysical, biochemical, and physiological traits using non-destructive imaging and sensor-derived time-series data (Rebetzke et al., 2019; Roitsch et al., 2019; Grzybowski et al., 2021). Currently, rapid progress has been made in the plant phenotyping field based on quantifying traits of interest using hyperspectral imaging (HSI). HSI has been used to estimated biophysical traits, such as plant height and biomass (Aasen et al., 2015; Yue et al., 2017); biochemical traits, such as water content, chlorophyll, and nitrogen (Quemada et al., 2014; Zhou et al., 2018; Xie and Yang, 2020); physiological traits, such as salt, heat, and drought stress tolerance and photosynthesis (Zarco-Tejada et al., 2013; Mo et al., 2017). HSI is a non-destructive, rapid, remotely sensed method of plant phenotyping that simultaneously extracts spectral and spatial information relevant to the overall plant heath. Hyperspectral images contain hundreds and even thousands of continuous wavebands in visible near-infrared (Vis–NIR) range, and thus the spectral information obtained is rich. However, image processing is complex and the spectra contain redundant information (Xie and Yang, 2020).

Lettuce (*Lactuca sativa* L. var. *longifolia*) is one of the most popular vegetables in the world, rich in vitamins, carotenoids, dietary fiber, and other trace elements (Kim et al., 2016; Xin et al., 2020). Moreover, the soluble solids content (SSC) and pH in biochemical traits are key indicators of lettuce taste and harvest time, and thus it is crucial in the lettuce growing industry (Eshkabilov et al., 2021). Due to the rising demand for lettuce in recent years and the tendency of this crop to lose moisture in a short time at room temperature, rapid strategies for lettuce quality assessment are needed (Mo et al., 2015; De Corato, 2020; Eshkabilov et al., 2021). Lettuce quality is evaluated based on nutrient content, appearance, and shelf life (Eshkabilov et al., 2021). The traditional evaluation methods are mainly visual and destructive. Moreover, these methods require specialists and are time-consuming and costly (Simko et al., 2018; Simko and Hayes, 2018). Therefore, it is important to develop a method that can rapidly and non-destructively assess lettuce quality in phenotyping traits analysis.

Predictive modeling of some lettuce phenotyping traits has been developed using spectral reflectance obtained *via* hyperspectral technique combined with machine learning methods. Eshkabilov et al. (2021) detected the nutrient content of lettuces using partial least squares regression (PLSR) and principal component analysis (PCA). Wavelet transform (WT) and PLSR have been used to assess the moisture content in lettuce leaves (Zhou et al., 2018). Also, ANOVA, artificial neural networks (ANN), competitive adaptive reweighed sampling (CARS), random forest (RF), successive projections algorithm (SPA), and least squares support vector regression (LSSVR) have been used to study the responses of lettuce to biotic and abiotic stresses, such as worms, water and pesticide (Mo et al., 2017; Osco et al., 2019). Most studies have focused on the leaf scale of lettuces, with only a few focusing on the canopy scale of lettuces. However, high-throughput plant phenotyping (HTPP) platforms can extract canopy scale features (Nyonje et al., 2021). Besides, some studies had small sample sizes of less than 100 and only one cultivar (Mo et al., 2015, 2017; Sun et al., 2018; Eshkabilov et al., 2021). The spectra of lettuces are distinguishing between different varieties, and for the same variety of lettuces, the spectra are also distinguishing in different growth states. Therefore, the insufficient sample size and number of cultivars can compromise the robustness of the established models used to predict plant phenotyping traits. Additionally, raw spectra used to develop models contain noise and redundant information, limiting their application in HTPP platforms. As a result, pretreatment and wavelength selection are usually conducted using multivariate analysis methods before modeling (Gao et al., 2021). The spectra obtained by different pretreatment methods have a great influence on the modeling, and sometimes even have negative effects. Therefore, the application of multivariate analysis method to the HTPP platforms may reduce the throughput and prediction accuracy.

Recent advances in machine learning have shown that deep learning algorithms can automatically learn to extract features from raw data and significantly improve modeling performance for many spectral analysis tasks (Singh et al., 2018; Kanjo et al., 2019; Rehman et al., 2020). Sun et al. (2019, 2021) employed a deep brief network to estimate cadmium and lead contents of lettuces with high accuracy. Rehman et al. (2020) also developed a modified Inception module to predict the relative water content (RWC) of maize leaves and achieved a determination coefficient (*R*^2^) of 0.872 for RWC. Aich and Stavness (2017) used deep convolutional and deconvolutional networks for leaf counting and obtained mean and standard deviation of absolute count difference of 1.62 and 2.30. Wang et al. (2019) developed the SegRoot model based on convolutional neural networks (CNN) to segment root from complex soil background with *R*^2^ of 0.9791. Wang et al. (2017) employed deep VGG16 model to evaluate apple black rot with accuracy of 90.4%. Deep learning models include CNN model and fully connected neural networks (FCNN) model (Furbank et al., 2021). The purposes of this study are: (1) two end-to-end deep learning models were developed for rapid and non-destructive prediction of phenotyping traits of lettuce canopy in hyperspectral applications; (2) the proposed models can directly use the raw reflectance spectra as input to obtain prediction for biochemical traits, such as SSC and pH in lettuce phenotyping traits; and (3) to compare the difference in performance between the models built by various linear and nonlinear multivariate analysis methods and deep learning models. Specifically, the developed FCNN model (DeepFC) and the two-dimensional CNN model (Deep2D) could directly use the raw average spectral reflectance as input to predict the SSC and pH of lettuce canopy. The models could automatically learn to better extract the abstract features related to SSC and pH and thus did not require pretreatment or dimensionality reduction. Our models predicted SSC and pH better than multivariate analysis methods and were suitable for application in the HTPP platforms.

## Materials and Methods

### Plant Materials

**Figure 1:**
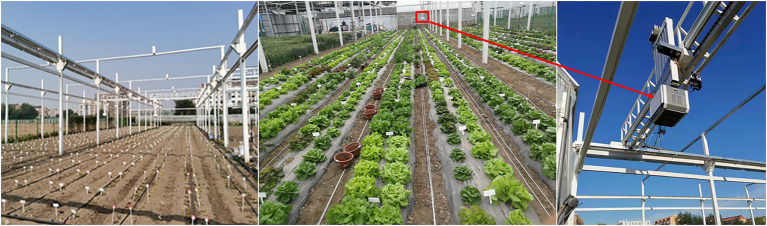
Picture of plants and LQ-FieldPheno in the field.

In this study, three annual bolting lettuce cultivars (Butter, Leaf, and Roman) with fast-growing and excellent quality were planted under open field conditions at the Research Center of Information Technology, Beijing Academy of Agriculture and Forestry Sciences (39.9438°N, 116.2876°E) on April 12, 2021. A field HTPP platform (LQ-FieldPheno, Beijing, China) was deployed at the experimental site (Figure 1). The plants were grown on raised beds with furrows between the beds (Figure 1). The growing environments of plants are: (1) no fertilizers were applied to the soil; (2) drip irrigation was conducted under professional supervision; and (3) water treatment was the same for all plants. Forty-five lettuces were selected from each cultivar on May 15, 20, and 25, 2021, put into the flowerpots, and then taken to the laboratory near the field for HSI. Each lettuce was imaged, then the lettuce juice was obtained as follows: (1) the lettuce roots were removed to obtain the leaves; (2) a hand-crank juicer (LKM-ZZ01, Like-me Technology Co., Ltd., China) was then used to extract the juice, finally store (3) in a centrifugal tube (capacity: 50 ml). A total of 387 lettuce juices were finally analyzed since the labels of eighteen lettuce samples were lost during the experiment (Supplementary Table S1). A digital refractometer (PAL-1, ATAGO Co., Ltd., Japan) and a pH meter (206pH1, Testo, Germany) were used to measure SSC and pH of lettuce juices, respectively.

### Hyperspectral Imaging System

The Vis-NIR hyperspectral images of lettuce plants were acquired in reflectance mode in a black room (length: 1.5 m, width: 1.5 m, and height: 2.5 m). The HSI system consists of the following modules: (1) a high spectrograph (GaiaField-V10E, Dualix Spectral imaging, China), (2) a CCD camera, (3) four tungsten halogen lamps, and (4) a computer (Ins 15-7,510-R1645S, Dell, United States) with image acquisition software (Specview, Dualix Spectral imaging, China). The spectrograph is a built-in push-broom style line-scanning and has a spectral range of 400–1,000 nm, with 256 spectral bands at a spectral resolution of 2.8 nm. The CCD camera has a spatial resolution of 696 pixels per scan line and was equipped with a 23 mm lens. The lamps provide 350–2,500 nm light with a power of 50 W. The distance between the lettuce plants and lens was set to 1 m, and the angle between the lamp and camera was set at 45° to provide enough light to the imaging area for image acquisition. The images (dimension: 775 × 696 × 256) were obtained at the exposure time, frame rate, gain, spatial binning, and spectral binning of 30 millisecond, 14 frames per second, 1, 2, and 4, respectively.

### Image Processing

The obtained raw hyperspectral images were distorted since the imaging mode of the hyperspectral system was set at push-broom style, and the non-planar camera lens and spectrometer were separated. As a result, the raw hyperspectral images were subjected to lens correction using the lens correction function provided by Specview. The corrected images were then calibrated to remove uneven light distribution and dark current from the sensor (Mishra et al., 2019; Gao et al., 2021). A 99% reflectivity flat polyvinyl chloride (PVC) board was scanned as the white reference to calibrate the light changes in the images, and the dark current from the hyperspectral sensor was removed by collecting the dark reference. The following calibration formula was used:


(1) $$R=\frac{R_{\mathrm{raw}}-R_{d}}{R_{w}-R_{d}}$$

*R*, *R*_raw_, *R*_w_, and *R*_d_ represent the calibrated image, raw hyperspectral image, white reference image acquired from the PVC board with 99% reflectance, and dark reference image obtained through shutting the lamps and covering the camera lens, respectively.

**Figure 2:**
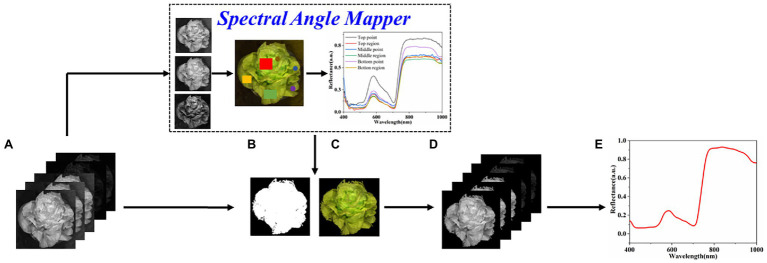
The process of plant spectra extraction. **(A)** Calibrated hyperspectral images, **(B)** binary plant mask, **(C)** masked RGB plant, **(D)** 256 bands corresponding to the ROI, and **(E)** extracted plant mean reflectance spectrum.

The difference in plant height causes significant differences in spectral reflectance and brightness in different plant parts. Common threshold segmentation method cannot adequately extract the region of interest (ROI) of the entire canopy. In this study, the mask of the ROI was obtained from the calibrated hyperspectral image using spectral angle mapper (SAM; Kim, 2021). SAM was conducted based on six spectra extracted from the top point, top region, middle point, middle region, bottom point, and bottom region of plant (Figure 2) *via* ENVI 5.3 software (ITT Visual Information Solutions, Boulder, CO, United States). The mask was multiplied using the calibrated image to obtain the ROI image. The mean spectrum was calculated by averaging the spectra of all pixels in the ROI images for further modeling analysis.

### Architecture of Proposed Models

Deep learning models are beneficial in the HTPP field due to their excellent information mining ability (Wang et al., 2019). For instance, it can be used for leaf counting (Aich and Stavness, 2017), image segmentation (Song et al., 2020), and quantitative or qualitative analysis (Wang et al., 2017; Kerkech et al., 2020). However, the deep learning model and spectroscopy combination are rarely used to quantify plant phenotyping traits. Herein, the models based on the two networks (2DCNN and FCNN) were developed for SSC and pH analysis of lettuces to understand the prediction accuracy of different network models for plant phenotyping traits.

**Figure 3:**
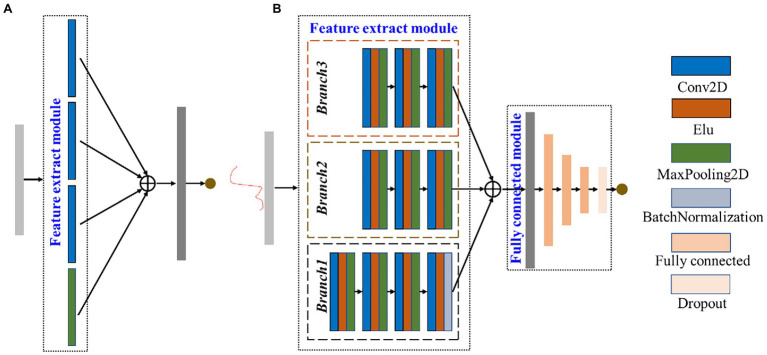
Architecture of the naïve version Inception module **(A)** and Deep2D **(B)**.

The CNN emulates the visual perceptual mechanisms of living things (Fu et al., 2020). CNN can learn grid-like topology features, such as pixels and audio. The amount of calculation is small due to the sharing of convolution kernel parameters in the hidden layer and the sparseness of inter-layer connection makes, and thus CNN has a stable effect and no additional feature engineering requirements for data. In the previous investigation, the performance of the model with linear stacking of convolutional layers was poor, possibly due to the insufficient extraction of the raw spectral features. Herein, the Inception module improved on the naïve version was introduced (Figures 3A,B). The CNN and FCNN layers of Deep2D used ELU and linear activation functions, respectively. The optimizers, loss, and metrics of Deep2D were Adam, root mean square error (RMSE), and mean absolute error (MAE). The Inception module was introduced because: (1) the use of different size convolutional kernels implies different size perceptual fields enabling learning features at multiple scales; (2) the final concatenate operation can fuse features at multiple scales; and (3) the depth and width of the network are well balanced to prevent the network from falling into saturation. As shown in Figure 3B, three scale features were used, and then the features were subjected to a concatenation operation. The dimension of concatenated features was large, and thus fully connected module was used to reduce the feature dimension and improve the robustness of Deep2D.

**Figure 4:**
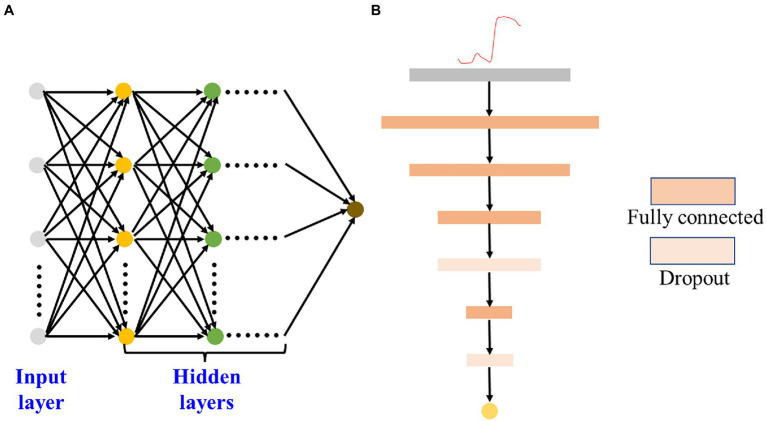
FCNN schematic chart **(A)** and architecture of the DeepFC **(B)**.

The FCNN model is a multi-layer perceptron (Scabini and Bruno, 2021). The principle of the perceptron is to find the most logical and robust hyperplane between classes. As shown in Figure 4A, unlike traditional perceptron, each node of the FCNN model has an operational relationship with all nodes in the next layer. FCNN usually has multiple hidden layers. Although adding hidden layers can better separate the data features, too many hidden layers can also increase the training time and produce overfitting. Herein, the dropout operation was introduced to prevent overfitting. The structure of the DeepFC is shown in Figure 4B, and the features in the input layer were first scaled up and then scaled down. The dropout operation was conducted on the top 2 layers after the inputting features. Moreover, the linear activation function was applied to hidden layers of DeepFC. The optimizers, loss, and metrics of DeepFC were the same as those of Deep2D.

### Comparison of Deep2D and DeepFC With Various Multivariate Analysis Methods

Three linear multivariate analysis methods [PLSR, locally weighted regression (LWR) and multiple linear regression (MLR)] and two nonlinear methods [ANN and support vector regression (SVR)] were used to establish models to compare with the proposed deep learning models (Deep2D and DeepFC).

PLSR is a multi-dependent variable *Y* against the multi-independent variable *X* modeling method. The method maximally extracts the principal components in *Y* and *X*, and maximizes the correlation between the principal components extracted from *X* and *Y* during the modeling process (Yu et al., 2015). Herein, *X* and *Y* represent the spectra and predicted values (SSC or pH), respectively. LWR is a nonparametric method for local regression analysis. It divides the samples into cells, performs polynomial fitting on the samples, and continuously repeats the process to obtain weighted regression curves in various cells. Finally, the centers of these regression curves are connected to form a complete regression curve (Raza and Zhong, 2019). MLR obtains a weighted summation relationship between each feature and the predicted values. The problems to be dealt with in practical work are usually complex multiple features, and thus compared with the univariate linear regression method, MLR is more suitable for use in practical work (Chung et al., 2021).

Artificial neural networks abstracts human brain neural networks based on information processing, thus establishing some simple models. Different networks are formed according to different connection methods. It is an operational model consisting of several interconnected nodes. Each node represents a specific output function (activation function). The connection between every two nodes represents a weight value for the signal passing through the connection (Osco et al., 2019). SVR is a key application branch of the support vector machine. It uses an optimal hyperplane that minimizes the total deviation of all sample points from the hyperplane, and then fits all the data through the optimal hyperplane (Zhang et al., 2017).

### Spectral Pretreatment and Wavelength Selection

Light inhomogeneity and background interference generate noise in the reflectance spectra extracted from the hyperspectral images. Moreover, there are pitfalls of wavelengths unrelated to SSC and pH and a high correlation between adjacent wavelengths in the high dimensional spectra. As a result, the accuracy of the models developed *via* multivariate analysis methods may be reduced. However, spectral pretreatment methods, such as moving window smoothing (MWS), Savitzky–Golay Filter (SG), first-order derivative (FDR), second-order derivative (SDR), and WT and wavelength selection including CARS may address these problems and improve the performance of the models.

MWS sets a smooth window that is moved over each spectrum to average the spectra, thus denoising spectra. SG is a filtering method based on a local polynomial least squares fit in the time domain. The most important characteristic of this filter is that the shape and width of the spectra can be ensured to be constant while filtering out the noise. Derivative spectra, such as FDR and SDR can effectively eliminate background interference and improves the signal-to-noise ratio of the spectra *via* derivation of the spectra. CARS uses the absolute value of the regression coefficient as index to weigh the importance of wavelengths, and can effectively select the optimal combination of wavelengths.

### Modeling and Model Evaluation

The reflectance spectra of 387 lettuces were divided into calibration and prediction sets with a ratio of 2:1 based on Kennard–Stone (KS) algorithm (Saptoro et al., 2012). A 15% proportion of the spectra from the calibration set was then randomly selected as the validation set. Specifically, the sample number of calibration, validation, and prediction sets were 219, 39, and 129, respectively. The SSC and pH values of 387 lettuces are shown in Table 1. For the models established *via* the multivariate analysis methods, the data sets were divided after pretreatment or wavelength selection. Spectra of 219 lettuces were used to develop the models, spectra of 39 lettuces were used to optimize the parameters of the models, and spectra of 129 lettuces were used to evaluate the performance of the established models.

**Table 1:** Reference measurement of SSC and pH of lettuces.

Phenotyping traits	Range	Mean	Variance
SSC (%)	0.8750–5.8250	3.1040	0.5667
pH	6.3125–6.8175	6.5945	0.0074

The performance of all established models was evaluated using the coefficient of determination and root mean square error for calibration set ( $R_{c}^{2}$ , RMSEC), validation set ( $R_{v}^{2}$ , RMSEV), and prediction set ( $R_{p}^{2}$ , RMSEP), and the relative percent difference of prediction set (RPD). The formulas for *R*^2^, RMSE, and RPD were as follows:


(2) $$R^{2}=1-\frac{\sum {\left(y_{p}-y_{r}\right)}^{2}}{\sum {\left(y_{p}-y\right)}^{2}}$$

(3) $$\mathrm{RMSE}=\sqrt{\frac{\sum {\left(y_{p}-y_{r}\right)}^{2}}{n}}$$

(4) $$RPD=\frac{SD}{\mathrm{RMSE}}$$

where *y_p_*, *y_r_*, and *y* are the prediction values, reference values, and mean value of reference values, respectively, and n is the number of samples, and SD is the standard deviation. The smaller the RMSEC, RMSEV, and RMSEP, the larger the $R_{c}^{2}$ , $R_{v}^{2}$ , $R_{p}^{2}$ , and RPD, and the better the performance of the model. The Deep2D and DeepFC were performed using Python 3.7.10 with TensorFlow 2.4.1 environment. The multivariate analysis methods, spectral pretreatment and wavelength selection were conducted using MATLAB2020 (Mathworks, Inc., United States). All experiments were conducted using DELL OptiPlex 7080 (Dell, Inc., United States) equipped with a 2.90 GHz Intel® Core™ i7-10700 processor, 32 GB of random-access memory, and an Nvidia Quadro P2200 graphical processing unit. The computer had Windows® 10 Home Edition 20H2 operation system.

## Results and Discussion

### Spectral Reflectance Signature

**Figure 5:**
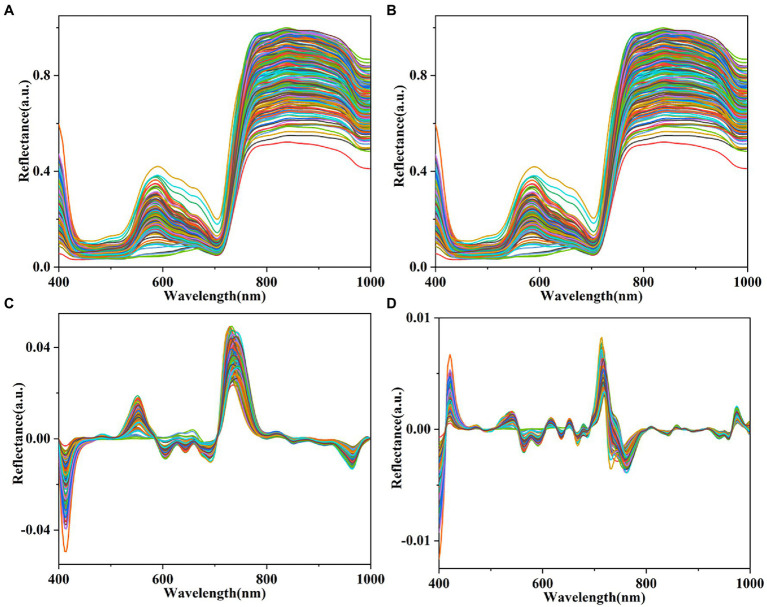
Mean spectral reflectance of plants. **(A)** Raw spectra and spectra pretreated by MWS **(B)**, FDR **(C)**, and SDR **(D)**.

The spectral reflectance of the lettuces in the wavelength ranges from 400 to 1,000 nm obtained from the hyperspectral images is shown in Figure 5A. The spectral reflectance signature of plants provides information on biophysical, physiological, and chemical features (Rehman et al., 2020). The band around 580 nm is related to xanthophylls (Pacumbaba and Beyl, 2011). The 710–760 nm (red-edge) band and band around 700 nm are related to chlorophyll (ElMasry et al., 2007; Pacumbaba and Beyl, 2011). The band around 980 nm in the NIR region is related to the O—H of water (Zhang et al., 2018). The spectral reflectance of the hyperspectral images of all lettuce plants pretreated using MWS, FDR, and SDR are shown in Figures 5B–D. Four bandwidth regions (400–435 nm, 515–650 nm, 690–780 nm, and 960–1,000 nm) of all plants have distinct variations. Herein, the reflectance values of MWS were not significantly different from the raw reflectance values. Compared with raw reflectance values, the variation of the reflectance values of FDR and SDR was significantly enhanced, especially SDR variation. A previous study also reported similar results (Eshkabilov et al., 2021).

**Figure 6:**
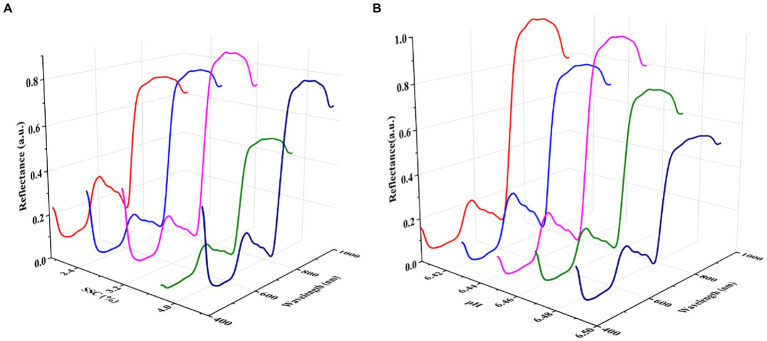
Mean spectral reflectance of plants of different SSC **(A)** and pH **(B)**.

The spectral reflectance of lettuces with different SSC and pH is shown in Figure 6. The reflectance values of lettuces with different SSC and pH had significant differences and changes. This phenomenon indicates that reflectance spectroscopy can be used to detect SSC and pH in lettuce. The spectral reflectance change at 600–800 nm was irregular with increasing SSC and pH. In the 400–500 nm and 800–1,000 nm regions, the spectral reflectance first increased, then decreased, and finally increased with increasing SSC. In contrast, the spectral reflectance change at the 800–1,000 nm region showed the opposite trend with increasing pH.

### Prediction Results of SSC and pH Based on Deep2D and DeepFC

The SSC and pH of lettuces were predicted using the regression models developed by Deep2D and DeepFC. The RMSE of the training, validation, and test sets were used as the evaluation criteria for the optimization of hyperparameters, such as batch size and learning rate. A batch size of 4 and a learning rate of 0.0001 provided a relatively lower RMSE and were selected to train the models. The detailed hyperparameters of the Deep2D and DeepFC are shown in Supplementary Tables S2, S3, and the analysis results of SSC and pH of lettuces are shown in Table 2.

**Table 2:** Prediction of SSC and pH using Deep2D and DeepFC.

Phenotyping traits	Models	$R_{c}^{2}$	RMSEC	$R_{v}^{2}$	RMSEV	$R_{p}^{2}$	RMSEP	RPD
SSC (%)	Deep2D	0.9642	0.1500	0.8974	0.1671	0.9030	0.1969	3.2237
	DeepFC	0.9031	0.2470	0.8527	0.2002	0.8385	0.2541	2.4980
pH	Deep2D	0.8842	0.0319	0.8241	0.0296	0.7807	0.0313	2.1445
	DeepFC	0.8670	0.0342	0.8674	0.0257	0.8490	0.0260	2.5839

**Figure 7:**
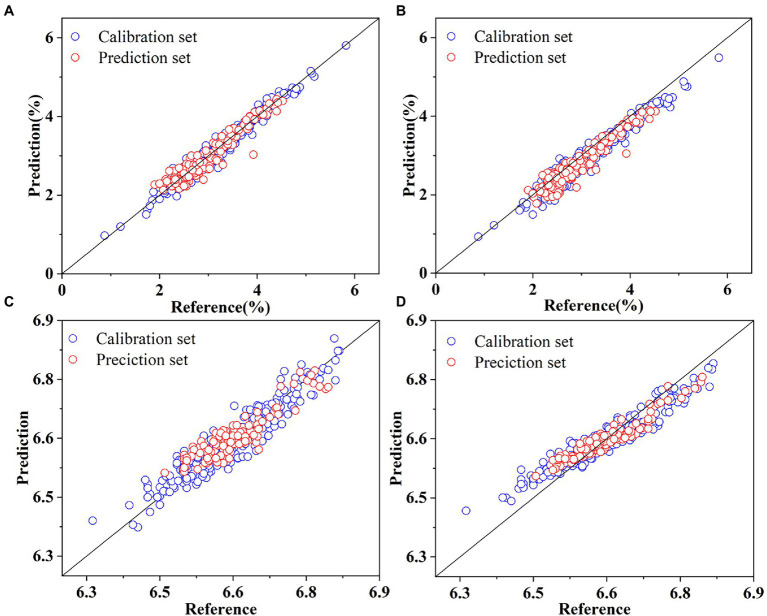
Prediction of SSC based on Deep2D **(A)** and DeepFC **(B)**, and pH based on Deep2D **(C)** and DeepFC **(D)**.

Deep2D obtained better results for SSC prediction than DeepFC ( $R_{c}^{2}$ : 0.9642, RMSEC: 0.1500, $R_{v}^{2}$ : 0.8974, RMSEV: 0.1671, $R_{p}^{2}$ : 0.9030, RMSEP: 0.1969, and RPD: 3.2237). Deep2D had excellent performance in predicting the SSC of lettuces since it had RPD greater than 3. This indicated that the multi-scale features associated with SSC extracted by Deep2D contain more abundant information than the single-scale features extracted by DeepFC. The robustness of Deep2D and DeepFC was similar in terms of the difference between $R_{c}^{2}$ and $R_{p}^{2}$ . However, the DeepFC had higher accuracy in analyzing pH ( $R_{c}^{2}$ : 0.8670, RMSEC: 0.0342, $R_{v}^{2}$ : 0.8674, RMSEV: 0.0257, $R_{p}^{2}$ : 0.8490, RMSEP: 0.0260, and RPD: 2.5839). The multi-scale features extracted by the Deep2D in relation to pH may have redundancy, and thus reduce the robustness of the model. DeepFC was more robust than Deep2D because it had a smaller difference between $R_{c}^{2}$ and $R_{p}^{2}$ . The prediction results for SSC and pH in the calibration and prediction sets based on the Deep2D and DeepFC are shown in Figure 7. The figures showed that the prediction values based on Deep2D were generally closer to the reference values for SSC. In the analysis of pH, for calibration set, the error of Deep2D was smaller, while for prediction set, the error of DeepFC was smaller. And the error of prediction set is more significant in actual application.

The two deep learning models had significantly different prediction abilities for various phenotyping traits. Therefore, a follow-up study should identify suitable network structures for prediction of different phenotyping traits. Moreover, a general network structure should be determined for simultaneous and accurate predictions of multiple phenotyping traits. Meanwhile, many studies have developed deep learning models combined with HSI to predict the phenotyping traits of plants and obtained good results. For instance, Rehman et al. (2020) used end-to-end deep model to predict the RWC of maize plants and obtained an $R_{p}^{2}$ of 0.872. Moreover, Zhou et al. (2020) detected heavy metals in lettuce using a stack convolution auto encoder ( $R_{p}^{2}$ : 0.9418, RMSEP: 0.04123, and RPD: 3.214). Sun et al. (2019) estimated cadmium content in lettuce leaves using deep brief network and obtained optimal performance ( $R_{p}^{2}$ = 0.9234, RMSEP = 0.5423, and RPD = 3.5894). Most previous studies were based on the leaf scale of lettuce and obtained satisfactory results. Although the phenotyping traits of lettuce canopy are also crucial in the actual production needs and consumer choices, the study of the lettuce canopy is rare. This study and the abovementioned studies have demonstrated that deep learning models combined with HSI techniques can significantly predict plant phenotyping traits.

### Prediction Results of SSC and pH Based on Various Multivariate Analysis Methods

Multivariate analysis methods, such as PLSR, LWR, MLR, ANN, and SVR were used to analyze the SSC and pH of the lettuces to more intuitively understand the performance of the Deep2D and DeepFC. Compared with deep learning models, multivariate analysis methods had much less ability to identify noise and effective information in spectra. As a result, multiple pretreatment methods (MWS, SG, FDR, SDR, and WT) were used to pretreat the spectra to reduce these negative effects before building regression models using multivariate analysis methods. The prediction results of SSC and pH of lettuces are shown in Tables 3, 4, and the corresponding parameter settings are shown in Supplementary Tables S4, S5.

**Table 3:** Prediction of the SSC of lettuces based on various multivariate analysis methods.

Phenotyping trait	Models	Pretreatment	$R_{c}^{2}$	RMSEC	$R_{v}^{2}$	RMSEV	$R_{p}^{2}$	RMSEP	RPD
SSC (%)	PLSR	Raw	0.9471	0.1846	0.9296	0.1677	0.8170	0.2658	2.3464
		MWS	0.9274	0.2163	0.9274	0.1703	0.8346	0.2527	2.4684
		SG	0.9284	0.2149	0.9212	0.1775	0.8404	0.2483	2.5126
		FDR	0.9246	0.2203	0.8542	0.2621	0.8400	0.2556	2.5098
		SDR	0.9465	0.1826	0.9332	0.1895	0.8581	0.2510	2.6648
		WT	0.9370	0.2014	0.9169	0.1822	0.8365	0.2512	2.4831
	LWR	Raw	0.9358	0.2034	0.9125	0.1870	0.8273	0.2582	2.4159
		MWS	0.9288	0.2142	0.9219	0.1767	0.8348	0.2526	2.4698
		SG	0.9278	0.2157	0.9106	0.1890	0.8389	0.2494	2.5012
		FDR	0.9178	0.2300	0.8403	0.2743	0.8387	0.2566	2.4999
		SDR	0.9183	0.2209	0.9249	0.2040	0.8587	0.2657	2.6705
		WT	0.9210	0.2255	0.8835	0.2158	0.8183	0.2648	2.3552
	MLR	Raw	0.9683	0.1430	0.9480	0.1442	0.7063	0.3367	1.8524
		MWS	0.9350	0.2046	0.9314	0.1657	0.8062	0.2735	2.2803
		SG	0.9399	0.1968	0.9293	0.1681	0.8056	0.2740	2.2769
		FDR	0.9537	0.1727	0.9333	0.1773	0.8055	0.2818	2.2763
		SDR	0.9476	0.1801	0.9320	0.1987	0.8148	0.2910	2.3327
		WT	0.9542	0.1718	0.9396	0.1554	0.8072	0.2728	2.2865
	ANN	Raw	0.9113	0.2391	0.8549	0.2409	0.7889	0.2855	2.1849
		MWS	0.8913	0.2646	0.8376	0.2548	0.7763	0.2939	2.1227
		SG	0.9014	0.2520	0.8633	0.2338	0.7864	0.2872	2.1722
		FDR	0.9584	0.1615	0.9473	0.1614	0.8170	0.2794	2.3468
		SDR	0.9627	0.1516	0.9532	0.1583	0.8170	0.2908	2.3470
		WT	0.9266	0.2175	0.8912	0.2086	0.7968	0.2801	2.2270
	SVR	Raw	0.7186	0.4259	0.7142	0.3397	0.6615	0.3658	1.7053
		MWS	0.6838	0.4552	0.6839	0.3557	0.5721	0.4150	1.5030
		SG	0.7073	0.4367	0.7172	0.3501	0.5868	0.4075	1.5308
		FDR	0.8230	0.3391	0.7343	0.3031	0.8009	0.2921	2.2045
		SDR	0.8768	0.2799	0.8893	0.2635	0.7928	0.3004	2.1765	
		WT	0.7121	0.4337	0.6990	0.3491	0.5835	0.4148	1.5036

**Table 4:** Prediction of the pH of lettuces based on various multivariate analysis methods.

Phenotyping trait	Models	Pretreatment	$R_{c}^{2}$	RMSEC	$R_{v}^{2}$	RMSEV	$R_{p}^{2}$	RMSEP	RPD
pH	PLSR	Raw	0.8617	0.0344	0.7778	0.0342	0.7092	0.0373	1.8623
		MWS	0.8667	0.0338	0.8105	0.0316	0.7092	0.0373	1.8624
		SG	0.8753	0.0327	0.8170	0.0310	0.7205	0.0366	1.8996
		FDR	0.8881	0.0309	0.8714	0.0216	0.6884	0.0395	1.7990
		SDR	0.8828	0.0314	0.7105	0.0322	0.6635	0.0419	1.7312
		WT	0.8840	0.0315	0.8482	0.0283	0.7259	0.0363	1.9180
	LWR	Raw	0.8696	0.0334	0.8378	0.0326	0.7274	0.0362	1.9233
		MWS	0.8734	0.0329	0.8511	0.0313	0.7380	0.0354	1.9619
		SG	0.8646	0.0341	0.8194	0.0344	0.7309	0.0359	1.9357
		FDR	0.8782	0.0324	0.8682	0.0239	0.7120	0.0375	1.8711
		SDR	0.8651	0.0334	0.7783	0.0372	0.7355	0.0381	1.9525
		WT	0.8740	0.0329	0.8667	0.0296	0.7368	0.0355	1.9574
	MLR	Raw	0.9434	0.0220	0.9025	0.0227	0.6182	0.0472	1.5162
		MWS	0.8811	0.0321	0.8342	0.0341	0.6692	0.0393	1.7460
		SG	0.8780	0.0323	0.8305	0.0299	0.6976	0.0381	1.8262
		FDR	0.8879	0.0308	0.8080	0.0278	0.6665	0.0415	1.7389
		SDR	0.8831	0.0306	0.8663	0.0317	0.6768	0.0442	1.7665
		WT	0.9437	0.0220	0.9108	0.0217	0.6317	0.0447	1.6013
	ANN	Raw	0.9115	0.0275	0.8375	0.0332	0.6695	0.0398	1.7469
		MWS	0.8723	0.0331	0.7958	0.0366	0.6569	0.0406	1.7145
		SG	0.9090	0.0279	0.8476	0.0316	0.6724	0.0396	1.7546
		FDR	0.8866	0.0313	0.7910	0.0317	0.5900	0.0443	1.5683
		SDR	0.8802	0.0318	0.8801	0.0274	0.6654	0.0419	1.7362
		WT	0.9253	0.0253	0.9087	0.0245	0.6656	0.0400	1.7367
	SVR	Raw	0.7629	0.0455	0.5847	0.0479	0.6618	0.0421	1.6508
		MWS	0.7496	0.0464	0.5473	0.0504	0.6515	0.0424	1.6380
		SG	0.7556	0.0455	0.6859	0.0420	0.6257	0.0453	1.5703
		FDR	0.8505	0.0361	0.8486	0.0246	0.7084	0.0386	1.7832
		SDR	0.8814	0.0321	0.7906	0.0359	0.6624	0.0410	1.6856	
		WT	0.7761	0.0440	0.6885	0.0416	0.6267	0.0443	1.5687

Compared with the other reflectance spectra, the FDR and SDR-pretreated spectra obtained better results for the models established by different multivariate analysis methods for SSC analysis, possibly because FDR and SDR enhanced the differences between the spectra of different SSC. All the pretreatment methods improved the precision of MLR models, and SDR obtained the best results with $R_{c}^{2}$ of 0.9476, RMSEC of 0.1801, $R_{v}^{2}$ of 0.9320, RMSEV of 0.1987, $R_{p}^{2}$ of 0.8148, RMSEP of 0.2910 and RPD of 2.3327. Compared with other models, SVR models had poor performance, with FDR spectra having $R_{p}^{2}$ > 0.8. The PLSR and LWR models had relatively great robustness. Moreover, LWR model with SDR spectra had the best results ( $R_{c}^{2}$ = 0.9183, RMSEC = 0.2209, $R_{v}^{2}$ = 0.9249, RMSEV = 0.2040, $R_{p}^{2}$ = 0.8587, RMSEP = 0.2657, and RPD = 2.6705). However, the best SSC results obtained by multivariate analysis methods were still lower than the results of Deep2D.

The ANN models had the worst performance for pH prediction. Compared with the raw spectra, only SG improved the accuracy of the models and obtained $R_{c}^{2}$ of 0.9090, RMSEC of 0.0279, $R_{v}^{2}$ of 0.8476, RMSEV of 0.0316, $R_{p}^{2}$ of 0.6724, RMSEP of 0.0396, and RPD of 1.7546. Although all pretreatment methods enhanced the robustness of MLR models, the $R_{p}^{2}$ corresponding to the best results was lower than 0.7. The PLSR and LWR models had relatively high performance. The results of PLSR, LWR, and MLR were similar to their results for SSC. Moreover, MWS, SG, and WT improved the accuracy of both PLSR and LWR models, and LWR model with MWS spectra had the best results ( $R_{c}^{2}$ : 0.8734, RMSEC: 0.0329, $R_{v}^{2}$ : 0.8511, RMSEV: 0.0313, $R_{p}^{2}$ : 0.7380, RMSEP: 0.0354, and RPD: 1.9619).

**Figure 8:**
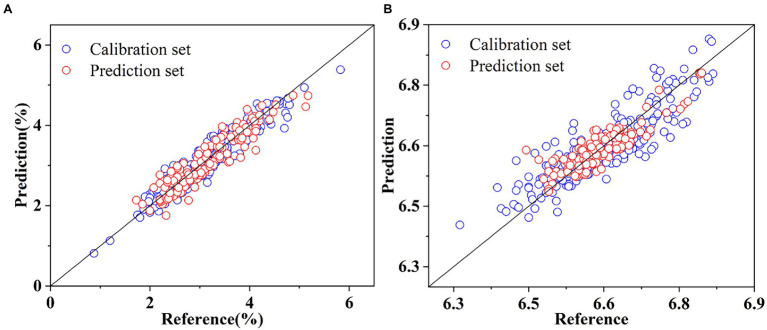
Prediction of SSC based on LWR and SDR **(A)**, and pH based on LWR and MWS **(B)**.

The results of the models established by multivariate analysis methods showed that the influence of different pretreatment methods on the same model was not always positive, and the effect of the same pretreatment method on different models was different. Therefore, it is important to select the appropriate multivariate analysis methods and pretreatment methods. Compared with the deep learning models, the manual feature engineering of multivariate analysis methods is indispensable before modeling, which reduces the throughput of data process in the HTPP platforms. The optimal prediction results for SSC and pH based on multivariate analysis methods are shown in Figure 8. The number of data points concentrated near the fitting line was significantly less than the optimal results in Figure 7. In summary, the performance of the models developed by multivariate analysis methods was significantly inferior to the performance of the proposed deep learning models. Therefore, deep learning algorithms can automatically learn to extract features from raw data and develop highly accurate models compared with multivariate analysis methods. Some previous studies also reported similar results (Singh et al., 2018; Kanjo et al., 2019; Rehman et al., 2020).

### Prediction Results of SSC and pH Based on CARS

The reflectivity spectra extracted from hyperspectral images have a narrow bandwidth and a high dimension, leading to a high correlation of adjacent wavelengths and the existence of information unrelated to SSC and pH in the wavelengths (Tian et al., 2018). As a result, this can decrease the precision of the models established *via* multivariate analysis methods and increase the computational complexity. Herein, CARS was used to select key wavelengths from the pretreated spectra to obtain relatively better results. Three preferred pretreatment methods were selected for wavelength selection (SG, FDR, and SDR for SSC and MWS, SG, and WT for pH). Models were then established based on the superior multivariate analysis methods (PLSR and LWR) to observe result changes compared with the full-range spectra.

The analysis results are shown in Table 5, and specific parameter settings and concrete selected wavelengths are shown in Supplementary Tables S6, S7. Only model based on LWR and SG had improved performance for SSC prediction ( $R_{c}^{2}$ : 0.9210, RMSEC: 0.2225, $R_{v}^{2}$ : 0.8865, RMSEV: 0.2233, $R_{p}^{2}$ : 0.8450, RMSEP: 0.2475, and RPD: 2.5400). Moreover, the number of variables decreased by about 90% after CARS. Compared with the performance of the model for the full-range spectra, the performance of the models slightly decreased based on the selected wavelengths. The PLSR models showed better performance for pH analysis based on wavelengths selected by CARS. The WT-pretreated spectra had the least wavelengths and better results ( $R_{c}^{2}$ : 0.7739, RMSEC: 0.0449, $R_{v}^{2}$ : 0.7589, RMSEV: 0.0331, $R_{p}^{2}$ : 0.7404, RMSEP: 0.0322, and RPD: 1.9626). Meanwhile, the LWR and WT model had lowest prediction errors ( $R_{c}^{2}$ = 0.7628, RMSEC = 0.0459, $R_{v}^{2}$ = 0.6461, RMSEV = 0.0382, $R_{p}^{2}$ = 0.7477, RMSEP = 0.0330, and RPD = 2.0532). Besides, only the LWR and WT model obtained an RPD > 2 among the pH prediction models based on multivariate analysis methods. However, DeepFC still outperformed the optimal multivariate analysis methods-based model. These results showed that CARS selected different number of bands and specific bands for different pretreated spectra and models. Therefore, compared with multivariate analysis methods-based models, deep learning algorithms reduce the time and failure rate of manual feature extraction and are thus more suitable for application in HTPP platforms.

**Table 5:** Prediction of SSC and pH combining with CARS.

Phenotyping traits	Models	Pretreatment	NVs	$R_{c}^{2}$	RMSEC	$R_{v}^{2}$	RMSEV	$R_{p}^{2}$	RMSEP	RPD
SSC (%)	PLSR	SG	33	0.9196	0.2274	0.9093	0.1940	0.8378	0.2409	2.4831
		FDR	27	0.8794	0.2791	0.8474	0.2401	0.8397	0.2458	2.4977
		SDR	30	0.8888	0.2667	0.8687	0.2467	0.8447	0.2529	2.5377
	LWR	SG	25	0.9210	0.2225	0.8865	0.2233	0.8450	0.2475	2.5400
		FDR	24	0.9203	0.2291	0.8934	0.2448	0.8370	0.2355	2.4772
		SDR	30	0.8755	0.2822	0.8602	0.2546	0.8504	0.2482	2.5858
pH	PLSR	MWS	42	0.7366	0.0488	0.7679	0.0337	0.7104	0.0340	1.8584
		SG	43	0.7320	0.0490	0.6380	0.0351	0.7249	0.0337	1.9065
		WT	36	0.7739	0.0449	0.7589	0.0331	0.7404	0.0332	1.9626
	LWR	MWS	50	0.7954	0.0429	0.6856	0.0380	0.7145	0.0339	1.8714
		SG	48	0.7756	0.0451	0.7157	0.0331	0.7277	0.0326	1.9165
		WT	41	0.7628	0.0459	0.6461	0.0382	0.7477	0.0330	2.0532

## Conclusion

In this study, two end-to-end deep learning models based on 2DCNN and FCNN were proposed to predict SSC and pH of lettuce canopy, supplementing the research on phenotyping traits prediction of lettuce canopy scale. In the previous studies on the phenotyping traits of lettuce, before inputting the spectra into the model, manual feature engineering, such as pretreatment and wavelength selection was required. However, the proposed model of this study can take as input the raw mean reflectance spectra extracted from the hyperspectral images, and then directly output the SSC and pH of the lettuce canopy. The performance of the proposed models was also compared with the performance of five multivariate analysis methods (PLSR, SVR, MLR, ANN, LWR). Various pretreatment methods (MWS, SG, FDR, SDR, and WT) were used to denoise the spectra, while CARS was used to remove the redundant variables in the spectra to improve the performance of the models based on these five methods.

The proposed Deep2D and DeepFC were superior to all multivariate analysis methods. Herein, the Deep2D predicted the best results for SSC ( $R_{p}^{2}$ : 0.9030, RMSEP: 0.1969, and RPD: 3.2237). In contrast, the DeepFC predicted the best results for pH ( $R_{p}^{2}$ : 0.8490, RMSEP: 0.0260, and RPD: 2.5839). Additionally, PCA was used in previous study to predict SSC and pH of lettuce with *R*^2^ of 0.88 and 0.81, respectively (Supplementary Table S8), and the performance of the proposed deep learning models was also better than PCA models. These results indicate that the proposed Deep2D and DeepFC do not require any pretreatment or dimensionality reduction since they can automatically extract the optimal features associated with SSC and pH from the raw reflectance spectra. Therefore, deep learning models can predict SSC and pH better than multivariate analysis methods, reducing the time and error rate of feature selection in the analysis of plant phenotyping traits. However, it is necessary to determine the corresponding suitable or general networks structure for better quantitative analysis of various phenotyping traits of plant.

In further study, it is necessary to understand change of plant phenotyping traits over time to determine the optimal harvest time. Also, there are differences in the morphology of different varieties of lettuce, and thus the image characteristics can be considered to be integrated into the input of the proposed models to observe the performance of the models.

## Data Availability Statement

The original contributions presented in the study are included in the article/Supplementary Material, further inquiries can be directed to the corresponding authors.

## Author Contributions

SY, JF, and SS designed the research. SY, JF, XL, and WW conducted the experiments. SY analyzed the data and wrote the manuscript. SY and JF revised the manuscript. JF, XG, and CZ obtained project funding. All authors contributed to the article and approved the submitted version.

## Funding

This research was funded by the Construction of Collaborative Innovation Center of Beijing Academy of Agricultural and Forestry Sciences (KJCX201917), Beijing Nova Program (Z211100002121065), and Science and Technology Innovation Special Construction Funded Program of Beijing Academy of Agriculture and Forestry Sciences (KJCX20210413).

## Conflict of Interest

The authors declare that the research was conducted in the absence of any commercial or financial relationships that could be construed as a potential conflict of interest.

## Publisher’s Note

All claims expressed in this article are solely those of the authors and do not necessarily represent those of their affiliated organizations, or those of the publisher, the editors and the reviewers. Any product that may be evaluated in this article, or claim that may be made by its manufacturer, is not guaranteed or endorsed by the publisher.
