# Supplementary material for: Hyperspectral Technique Combined With Deep Learning Algorithm for Prediction of Phenotyping Traits in Lettuce
Source: Front Plant Sci. 2022 Jun 30;13:927832. doi: 10.3389/fpls.2022.927832 (PMC9279906; doi:10.3389/fpls.2022.927832)
Supplement: Supplementary file 1 [file Data_Sheet_1.docx]

**Hyperspectral technique combined with deep learning algorithm for prediction of phenotyping traits in lettuce**

Shuan Yu^1, 2, †^, Jiangchuan Fan^2, †^, Xianju Lu^2^, Weiliang Wen^2^, Song Shao^1, 2^, Xinyu Guo^2, *^, Chunjiang Zhao^1, 2, *^

^1^ *National Engineering Research Center for Agro-Ecological Big Data Analysis & Application, Anhui University, 111 Jiulong Road, Hefei, 230601, China,* ^2^ *Beijing Key Laboratory of Digital Plant, China National Engineering Research Center for Information Technology in Agriculture, Beijing, 100097, China*

* Correspondence: Xinyu Guo: guoxy@nercita.org.cn; Chunjiang Zhao: zhaocj@nercita.org.cn.

† These authors contributed equally to this work.

**Supplementary material**

The specific number of samples discarded by each cultivar due to label loss during the experiment is shown in Table S1. The final number of samples used for modelling were 125, 127, 135 for Butter, Leaf, and Roman cultivars, respectively.

**Table S1.** Samples lost during the experiment

| Cultivars | May 15, 2021 | May 20, 2021 | May 25, 2021 |
| --- | --- | --- | --- |
| Butter | - | 4 | 6 |
| Leaf | 6 | 2 | - |
| Roman | - | - | - |

Hyperparameter settings for Deep2D are shown in Table S2.

**Table S2**. Configuration of Deep2D

| Branch1 | Branch2 | Branch3 | Dense module |
| --- | --- | --- | --- |
| Convolution layer 64@1×3 | Convolution layer 64@1×3 | Convolution layer 64@1×1 | Flatten 20480 |
| MaxPooling layer 2 | MaxPooling layer 2 | MaxPooling layer 2 | Dense layer 512 |
| Convolution layer 128@1×3 | Convolution layer 64@1×3 | Convolution layer 64@1×1 | Dense layer 256 |
| MaxPooling layer 2 | MaxPooling layer 2 | MaxPooling layer 2 | Dense layer 128 |
| Convolution layer 256@1×3 | Convolution layer 64@1×3 | Convolution layer 64@1×1 | Dropout 0.5 |
| MaxPooling layer 2 | MaxPooling layer 2 | MaxPooling layer 2 |  |
| Convolution layer 512@1×3 |  |  |  |

Optimizer: ‘Adam’, loss: ‘RMSE’, batch size = 4, epochs = 2000, learning rate = 0.0001

batch size — the number of training data sent into the network for each training.

Epochs — the number of all data is sent into the network to complete a training session.

Hyperparameter settings for DeepFC are shown in Table S3.

**Table S3.** Configuration of DeepFC

| Model | Hyperparameters |
| --- | --- |
| DeepFC | Dense layer 512 |
|  | Dense layer 256 |
|  | Dense layer 128  Dropout 0.1 |
|  | Dense layer 64 |
|  | Dropout 0.1 |

Optimizer: ‘Adam’, loss: ‘RMSE’, batch size = 4, epochs = 1000, learning rate = 0.0001

Parameter settings for multivariate analysis methods, pretreatment methods, and CARS used in Tables 3-5 are shown in Tables S4-6.

**Table S4.** Parameter setting of various models in Table 3

| Models | Settings of parameters |
| --- | --- |
| PLSR | nLVs = 10 |
|  | nLVs = 10 |
|  | nLVs = 12 |
|  | nLVs = 11 |
|  | nLVs = 11 |
|  | nLVs = 11 |
| LWR | a = 86, n_neigh = 258, distance = 1, weighting = 1 |
|  | a = 75, n_neigh = 232, distance = 1, weighting = 1 |
|  | a = 73, n_neigh = 229, distance = 1, weighting = 1 |
|  | a = 74, n_neigh = 258, distance = 1, weighting = 1 |
|  | a = 75, n_neigh = 258, distance = 1, weighting = 1 |
|  | a = 79, n_neigh = 258, distance = 1, weighting = 1 |
| MLR | intercept = 1 |
|  | intercept = 1 |
|  | intercept = 1 |
|  | intercept = 1 |
|  | intercept = 1 |
|  | intercept = 1 |
| ANN | topo = [‘HH’, ‘L-’], epochs = 40 |
|  | topo = [‘HH’, ‘L-’], epochs = 40 |
|  | topo = [‘HH’, ‘L-’], epochs = 40 |
|  | topo = [‘HH’, ‘H-’], epochs = 40 |
|  | topo = [‘HH’, ‘H-’], epochs = 25 |
|  | topo = [‘HH’, ‘H-’], epochs = 45 |
| SVR | linear kernel function, p = 0.35, c = 2048, g = 0.00097656 |
|  | linear kernel function, p = 0.5, c = 2048, g = 0.00048828 |
|  | linear kernel function, p = 0.5, c = 16384, g = 0.00019531 |
|  | linear kernel function, p = 0.5, c = 16384, g = 0.0019531 |
|  | linear kernel function, p = 0.4, c = 16384, g = 0.0019531 |
|  | linear kernel function, p = 0.4, c = 16384, g = 0.00097656 |

The settings of pretreatment methods:

MWS: width of windows = 5

SG: number of points = 15, polynomial order = 2, derivative = 0

FDR: number of points = 15, polynomial order = 2, derivative = 1

SDR: number of points = 15, polynomial order = 2, derivative = 2

WT: type of wavelet: ‘harr’, the layer number of transform = 1

PLSR: nLVs — number of latent variables.

LWR: a — dimensions to consider;

n_neigh — nearest neighbours to consider;

distance — 1=Euclidean, 2=Mahalanobis;

weighting — 1=Uniform, 2=Cubic.

MLR: intercept — 0=don't compute the intercept, 1=compute the intercept

ANN: topo — activation function, H=hyperbolic tangent, L=linear

SVR: p — value of loss function;

c — loss function parameters;

g — gamma function in the kernel function.

**Table S5.** Parameter setting of various models in Table 4

| Models | Settings of parameters |
| --- | --- |
| PLSR | nLVs = 11 |
|  | nLVs = 13 |
|  | nLVs = 13 |
|  | nLVs = 13 |
|  | nLVs = 12 |
|  | nLVs = 12 |
| LWR | a = 59, n_neigh = 208, distance = 1, weighting = 1 |
|  | a = 63, n_neigh = 196, distance = 1, weighting = 1 |
|  | a = 63, n_neigh = 191, distance = 1, weighting = 1 |
|  | a = 61, n_neigh = 160, distance = 1, weighting = 1 |
|  | a = 68, n_neigh = 206, distance = 1, weighting = 1 |
|  | a = 80, n_neigh = 231, distance = 1, weighting = 1 |
| MLR | intercept = 1 |
|  | intercept = 1 |
|  | intercept = 1 |
|  | intercept = 1 |
|  | intercept = 1 |
|  | intercept = 1 |
| ANN | topo = [‘HH’, ‘H-’], epochs = 40 |
|  | topo = [‘HH’, ‘H-’], epochs = 35 |
|  | topo = [‘HH’, ‘H-’], epochs = 40 |
|  | topo = [‘HH’, ‘L-’], epochs = 20 |
|  | topo = [‘HH’, ‘L-’], epochs = 15 |
|  | topo = [‘HH’, ‘L-’], epochs = 45 |
| SVR | linear kernel function, p = 0.35, c = 1024, g = 0.0039063 |
|  | linear kernel function, p = 0.5, c = 1024, g = 0.0039063 |
|  | linear kernel function, p = 0.5, c = 1024, g = 0.0039063 |
|  | linear kernel function, p = 0.5, c = 1024, g = 0.0019531 |
|  | linear kernel function, p = 0.4, c = 512, g = 0.0019531 |
|  | linear kernel function, p = 0.4, c = 1024, g = 0.0039063 |

The settings of pretreatment methods are the same as in Table 3.

**Table S6.** Parameter setting of various models in Table 5

| Phenotyping traits | Models | Settings of parameters |
| --- | --- | --- |
| SSC (%) | PLSR | nLVs = 3 |
|  |  | nLVs = 3 |
|  |  | nLVs = 3 |
|  | LWR | a = 15, n_neigh = 231, distance = 1, weighting = 1 |
|  |  | a = 15, n_neigh = 231, distance = 1, weighting = 1 |
|  |  | a = 20, n_neigh = 231, distance = 1, weighting = 1 |
| pH | PLSR | nLVs = 4 |
|  |  | nLVs = 4 |
|  |  | nLVs = 4 |
|  | LWR | a = 8, n_neigh = 206, distance = 1, weighting = 1 |
|  |  | a = 8, n_neigh = 206, distance = 1, weighting = 1 |
|  |  | a = 5, n_neigh = 206, distance = 1, weighting = 1 |

The parameter settings of CARS: A = 9, fold = 5, method = ‘center’, num = 50

CARS: A — the maximal principle to extract;

fold — the group number for cross validation;

method — pretreatment method;

num — the number of Monte Carlo Sampling runs.

The concrete wavelengths selected using CARS are shown in Table S7, and the wavelength order is arranged in descending order of weight.

**Table S7.** Concrete wavelengths selected using CARS in Table 5

| Phenotyping traits | | Models | Pretreatment | Wavelengths (nm) |
| --- | --- | --- | --- | --- |
| SSC (%) | PLSR | SG | 419;415;1000;439;942;435;448;972;456;674;954;628;995;992;828;944;472;637;656;630;937;487;619;639;825;646;677;463;667;559;665;621;568 |  |
|  |  | FDR | 660;653;663;649;602;605;639;635;670;596;786;949;612;959;884;714;498;698;712;962;741;400;402;404;406;408;415 |  |
|  |  | SDR | 972;977;942;801;982;487;765;472;852;948;874;899;974;847;889;503;937;985;760;494;796;505;448;483;755;904;912;769;786;463 |  |
|  | LWR | SG | 419;415;1000;439;942;435;448;972;456;674;954;628;995;992;828;944;472;637;656;630;937;487;619;639;825 |  |
|  |  | FDR | 660;653;663;649;602;605;639;635;670;596;786;949;612;959; 884;714;498;698;712;962;741;400;402;404 |  |
|  |  | SDR | 972;977;942;801;982;487;765;472;852;948;874;899;974;847;889;503;937;985;760;494;796;505;448;483;755;904;912;769;786;463 |  |
| pH | PLSR | MWS | 600;602;612;621;632;609;463;591;487;490;593;614;501;532;619;639;476;811;498;808;510;598;521;651;912;644;474;860;512;584;503;748;485;523;589;658;818;472;830;635;729;653 |  |
|  |  | SG | 589;612;609;621;487;632;872;602;887;485;490;600;815;448;818;806;474;498;808;501;570;828;635;796;472;523;867;452;642;430;535;461;987;582;912;745;492;644;463;789;512;496;510 |  |
|  |  | WT | 600;681;677;548;525;972;644;705;691;487;490;501;503;729;609;612;589;719;543;498;703;474;476;724;580;818;820;755;630;568;656;658;698;738;483;485 |  |
|  | LWR | MWS | 600;602;612;621;632;609;463;591;487;490;593;614;501;532;619;639;476;811;498;808;510;598;521;651;912;644;474;860;512;584;503;748;485;523;589;658;818;472;830;635;729;653;736;496;441;705;432;492;580;957 |  |
|  |  | SG | 589;612;609;621;487;632;872;602;887;485;490;600;815;448;818;806;474;498;808;501;570;828;635;796;472;523;867;452;642;430;535;461;987;582;912;745;492;644;463;789;512;496;510;459;619;521;470;605 |  |
|  |  | WT | 600;681;677;548;525;972;644;705;691;487;490;501;503;729;609;612;589;719;543;498;703;474;476;724;580;818;820;755;630;568;656;658;698;738;483;485;463;922;924;862;864 |  |

Eshkabilov et al. (2021) employed PCA for prediction of lettuce SSC and pH with R^2^ of 0.88 and 0.81, respectively.

**Table S8.** Previous study on SSC and pH of lettuce using PCA

| Model | Phenotyping traits | R^2^ |
| --- | --- | --- |
| PCA | SSC | 0.88 |
|  | pH | 0.81 |
